# Functional evaluation of pancreatic islets from patients with Beckwith–Wiedemann syndrome and congenital hyperinsulinism

**DOI:** 10.1210/clinem/dgag050

**Published:** 2026-02-15

**Authors:** Christine A Juliana, Changhong Li, Jinghua Chai, Kara E Boodhansingh, Lauren M Mitteer, Jennifer M Kalish, Tricia R Bhatti, Nick Scott Adzick, Diva D De Leon

**Affiliations:** Division of Endocrinology and Diabetes, Children's Hospital of Philadelphia, Philadelphia, PA 19104, USA; Division of Endocrinology and Diabetes, Children's Hospital of Philadelphia, Philadelphia, PA 19104, USA; Department of Pediatrics, Perelman School of Medicine at the University of Pennsylvania, Philadelphia, PA 19104, USA; Division of Endocrinology and Diabetes, Children's Hospital of Philadelphia, Philadelphia, PA 19104, USA; Division of Endocrinology and Diabetes, Children's Hospital of Philadelphia, Philadelphia, PA 19104, USA; Division of Endocrinology and Diabetes, Children's Hospital of Philadelphia, Philadelphia, PA 19104, USA; Department of Pediatrics, Perelman School of Medicine at the University of Pennsylvania, Philadelphia, PA 19104, USA; Division of Human Genetics, Children's Hospital of Philadelphia, Philadelphia, PA 19104, USA; Department of Genetics, Perelman School of Medicine at the University of Pennsylvania, Philadelphia, PA 19104, USA; Department of Pathology, Children's Hospital of Philadelphia, Perelman School of Medicine at the University of Pennsylvania, Philadelphia, PA 19104, USA; Department of Surgery, Children's Hospital of Philadelphia, Perelman School of Medicine at the University of Pennsylvania, Philadelphia, PA 19104, USA; Division of Endocrinology and Diabetes, Children's Hospital of Philadelphia, Philadelphia, PA 19104, USA; Department of Pediatrics, Perelman School of Medicine at the University of Pennsylvania, Philadelphia, PA 19104, USA

**Keywords:** Beckwith–Wiedemann syndrome (BWS), insulin secretion, hyperinsulinism, hypoglycemia, pancreas, overgrowth

## Abstract

**Context:**

Beckwith–Wiedemann Syndrome (BWS) is an overgrowth syndrome caused by various genetic or epigenetic abnormalities in a cluster of imprinted genes on chromosome 11p15. Congenital hyperinsulinism (HI) is one of the cardinal features of BWS, but the pathophysiology of HI in BWS has not been clearly defined.

**Objective:**

We describe the islet phenotype of a series of infants with severe HI and comorbid BWS who required pancreatectomy for intractable hypoglycemia.

**Methods:**

The cases are a subset of HI patients who required pancreatectomy and had Beckwith–Wiedemann Syndrome. Molecular testing for BWS was performed by SNP array and chromosome 11p15 methylation analysis. Functional analysis of insulin secretion in pancreatic islets isolated from pancreatectomy samples was completed with perifusion experiments and cytosolic Ca^2+^ measurements.

**Results:**

Similar to what we had previously described in islets isolated from the pancreas of infants with HI due to inactivating mutations in the K_ATP_ channel, BWS–HI islets have elevated concentrations of cytosolic calcium and secrete insulin in response to stimulation with a physiologic mixture of amino acids. However, unlike K_ATP_HI islets, most BWS–HI islets retain responsiveness to stimulation with glucose and the K_ATP_ channel inhibitor glyburide. Through RNAseq analysis, we observed that expression of *KCNQ1*, encoding the pore-forming subunit of a voltage-gated K + channel (Kv7.1), is reduced in BWS–HI islets compared to normal human islets (3-fold; *P* = 4.5 × 10-7).

**Conclusion:**

Our expression analysis and functional evaluation of islets isolated from BWS–HI patients reveal the spectrum of insulin secretion responses found across BWS etiologies and suggest a potential role for loss of *KCNQ1* expression in the complex pathophysiology responsible for the hyperinsulinism in BWS.

Beckwith–Wiedemann Syndrome (BWS) is the most common genetic overgrowth syndrome and is caused by various identified genetic or epigenetic abnormalities in a cluster of imprinted genes on human chromosome 11p15. The classically attributed features of BWS overgrowth include macroglossia, macrosomia, omphalocele, hemihypertrophy, and embryonal tumors (1). The genetic and epigenetic changes resulting in BWS generally occur early during development in a subset of cells that results in a mosaic mixture of both normal and BWS-affected cells and, thus, culminate in a spectrum of clinical forms and severities (2, 3). The level of mosaicism can be estimated, though testing of multiple tissues (eg, blood, skin, and pancreas) is necessary, as tissues can have different levels of loss of heterozygosity (LOH) of the chromosome 11p15 region (4, 5).

Chromosome 11p15.5 comprises 2 regions of imprinted genes in which expression is controlled by the methylation status of imprinting control regions 1 and 2 (IC1 and IC2). The regulated expression within these regions of the growth-promoting factor *IGF2*, tumor-suppressing long noncoding RNA *H19*, and the cell cycle regulator *p57/CDKN1C* establishes a delicate equilibrium between growth and inhibition of growth but also presents the opportunity for disruptive outcomes when expression levels are unbalanced. Normally, expression of *IGF2* and *H19* is mono-allelic and controlled by methylation at IC1. *IGF2* expression is paternally imprinted and occurs with methylation at IC1, while expression of *H19* is maternally imprinted and occurs when no methylation is present at IC1 (6, 7). Expression of *KCNQ1*, the pore-forming alpha subunit of the voltage-gated potassium channel Kv7.1, and *p57/CDKN1C* are maternally imprinted and require methylation at IC2 (8, 9). Expression of the long noncoding RNA *KCNQ1OT1*, which transcriptionally silences expression of *KCNQ1*, occurs on the antisense strand within the *KCNQ1* gene and is normally mono-allelically expressed from the paternal allele with no methylation at IC2 (10, 11). Several etiologies for dysregulated expression in these regions resulting in BWS have been identified and include: isolated hypomethylation at IC2 causing loss of expression of both *p57/CDKN1C* and *KCNQ1* (50% of cases); paternal isodisomy for chromosome 11p (pUPD11) leading to loss of *p57/CDKN1C* and *KCNQ1* with biallelic expression of *IGF2* (20% of cases); isolated hypermethylation at IC1 resulting in loss of *H19* and biallelic expression of *IGF2* (5-10% of cases); *p57/CDKN1C* maternal loss of function variants (5% of cases); and pathogenic copy number variants of the region (3% of cases) (2, 12). Patients with these epigenetic changes can exhibit a spectrum of effects with varying severity, ranging from classic BWS to more subtle overgrowth with hyperinsulinism (HI) or isolated overgrowth of a limb or organ (3, 13).

About 50% of infants with BWS have neonatal hypoglycemia due to hyperinsulinism (12), designating it as a cardinal feature of the syndrome (2). HI in BWS can be transient and mild, but, in some cases, the HI phenotype is as severe as HI due to inactivating mutations of the ATP-sensitive potassium channel (K_ATP_) and requires pancreatectomy for intractable hypoglycemia (13). K_ATP_ inactivating mutations in either of the bipartite subunits of the K_ATP_ channel, *ABCC8* or *KCNJ11*, have also been identified in some patients with BWS–HI, but are not necessary for the development of HI in these patients. Therefore, the mechanism of hyperinsulinism in BWS remains incompletely elucidated. While overgrowth of endocrine tissue, particularly beta cells, has been postulated as the cause of BWS–HI, the spectrum of phenotypes and the distinct functional changes that we have identified suggest an alternative mechanism. To address this dearth of understanding of the pathophysiology of HI in BWS, we assessed available patient data, functionally evaluated the changes in insulin secretion in pancreatic islets obtained from patients who required pancreatectomy, and examined gene expression changes in BWS–HI pancreatic islets using RNAseq.

## Research design and methods

Written informed consent was provided by all subjects or their parents. This study was approved by the Children's Hospital of Philadelphia (CHOP) Committees for the Protection of Human Subjects under protocol numbers 07-005772 and 08-006216.

### Patient cohort

The cases described here are a subset of HI patients seen at CHOP between 2011 and 2019 who required pancreatectomy and had Beckwith–Wiedemann Syndrome (13). The diagnosis of HI was based on previously described criteria: fasting hypoglycemia accompanied by inadequate suppression of plasma insulin, inappropriately low plasma free fatty acid and β-hydroxybutyrate concentrations, and an inappropriate glycemic response to glucagon at the time of hypoglycemia (14, 15). These cases were defined as being unresponsive to diazoxide, in that treatment with diazoxide at doses of up to 15 mg/kg/day was insufficient to reverse the cardinal feature of HI, hypoketotic hypoglycemia (16) and, therefore, they required surgical pancreatectomy to manage hypoglycemia. Mutation screening in genes known to be associated with HI was performed in commercial laboratories for all probands included in this study. Molecular testing for BWS was performed by SNP array and chromosome 11p15 methylation analysis on various tissue types including blood, skin, and pancreas (17).

### Islet isolation and perifusion

Fresh pancreatic surgical specimens from infants with HI were obtained at the time of pancreatectomy. The pancreatic tissue was injected with 0.75 mg/mL collagenase P (11249002001, Roche, Mannheim, Germany), minced with scissors, and then digested while shaking in a 37 °C water bath. Human islets were hand-picked using a dissection microscope and cultured in RPMI-1640 medium (11879, Gibco, USA) containing 5 mM glucose, 10% fetal bovine serum, 10% glutamine, and 10% penicillin/streptomycin for 2–3 days in a 5% CO_2_-humidified incubator temperature controlled to 37 °C (18).

For perifusion experiments, batches of cultured islets were loaded onto a nylon filter in a chamber and perifused with Krebs-Ringer bicarbonate buffer (19) at 37 °C. Islets were challenged with a glucose ramp (3 to 25 mM over 40 minutes), amino acid mixture (AAM) (20) ramp (0 to 12 mM over 40 minutes), glyburide (0.3 µM), diazoxide (200 µM), NN414 (5 µM), or KCl (30 mM) as denoted with fractions collected at 1 mL/min. Secreted insulin was measured by homogeneous time-resolved fluorescence (HTRF) (621N1PEH, CisBio, France) and read on a plate reader (CLARIOstar, BMG LABTECH, USA). Insulin secretion in response to stimuli was considered positive when insulin secretion increased by at least 50% above baseline levels.

### Cytosolic Ca^2+^ measurements

Cytosolic Ca^2+^ ([Ca^2+^]*_i_*) measurements using the ratio metric Ca^2+^-sensitive dye Fura-2AM (F1221, Invitrogen, Waltham, MA, USA) were conducted as described (21). Briefly, islets were preincubated with 3.3 µM Fura 2-AM in 0.33% DMSO in Krebs–Ringer bicarbonate buffer for 40 minutes at 37 °C. Next, islets were equilibrated in Krebs–Ringer bicarbonate buffer at 37 °C for 15 minutes before measurements, and then basal levels were measured before exposure to glucose (10 mM), AAM (4 mM), glyburide (0.3 µM), or KCl (30 mM). The ratio metric signals were obtained with excitation of 340 and 380 nm.

### RNAseq

RNA sequencing (RNAseq) analysis was completed on islets isolated from patients with BWS–HI (Cases 1, 2, 3, 4, and 7) and compared to that of islets isolated from age-matched controls from the normal section of pancreatectomies for focal HI. Total RNA was collected using the Qiagen AllPrep DNA/RNA Micro Kit (80284, Qiagen, USA) or Mini Kit (80204, Qiagen, USA). RNA quality was determined using the Agilent 2100 Bioanalyzer; RNA integrity numbers (RINs) were all >8. RNA-seq libraries were generated from 100 ng total RNA using the Illumina TruSeq Stranded Total RNA LT Sample Prep Kit (RS-122-2301, Illumina, USA). Libraries were single-end sequenced to 100 bp on an Illumina HiSeq 2500 System. Raw sequenced reads were filtered to retain only high-quality reads, and ribosomal reads and repeats were eliminated. Remaining reads were processed with RNA-Seq Unified Mapper (RUM), which aligns reads to the set of known transcripts included in RefSeq, UCSC known genes, and VEGA transcripts, and the genome, and outputs feature-level quantitation (transcript, exon, and intron). There were 176-193 million reads mapped to the genome for each BWS–HI case, while 36-48 million reads mapped to the genome for each control. To analyze global gene expression profiles, the number of uniquely aligning reads to mRNA transcripts in RefSeq and UCSC genes were extracted from the RUM output. Pairwise comparisons between groups were carried out using a custom script that implemented Bioconductor software's package edgeR to compute a *P*-value and fold-change for each transcript. The edgeR program adjusts for differences in sequencing depth across samples. The data were summarized for individual genes by selecting a “representative transcript” with the highest read counts. The resulting *P*-values were corrected for multiple testing using the Benjamini & Hochberg mode of the R function “*P*.adjust” to compute a false discovery rate (FDR). Transcripts were considered significantly differentially expressed if the FDR was less than 0.05.

### Statistics

Statistical analyses were performed with GraphPad Prism (v5-10; GraphPad Software, USA). Results are presented as mean ± standard deviation (SD).

## Results

We collected pancreatic surgical specimens to evaluate islet function from 11 cases of diazoxide-unresponsive BWS–HI that required surgical intervention. These infants were large for gestational age (LGA) at birth, presented with severe and persistent hypoglycemia shortly after birth and were diagnosed with hyperinsulinism as per standard clinical criteria (16). They had high glucose infusion rate (GIR) requirement of 10 to 25 mg/kg/min and were unresponsive to medical therapy with diazoxide requiring pancreatectomy for intractable hypoglycemia. The extent of pancreatectomy varied from 50% to 100%. Pancreatic histology was consistent with previously described features of BWS–HI (22), showing markedly increased endocrine tissue with an accentuation of the trabecular arrangement of islet cells.

### Molecular mechanism for BWS

Nearly all patients in this BWS–HI cohort (10 out of 11) were determined to have paternal uniparental isodisomy of the 11p15 chromosomal region (pUPD11) (Table 1, Cases 1-10). For 5 of these cases, no other HI-associated genetic variants were found, and the percentage of UPD identified ranged from 0% to 95% in blood, 0% to 30% in skin, and 80% to 85% in pancreas (Table 1). Four pUPD11 cases also had a paternally inherited pathogenic variant in *ABCC8* or *KCNJ11*, the genes encoding the beta cell ATP-sensitive potassium channel (Table 1, Cases 7-10). One pUPD11 case had a maternally inherited variant in *ABCC8* (Case 6). In all 5 cases, the UPD region included the *ABCC8* and *KCNJ11* loci. The paternally inherited variants in Cases 7, 9, and 10 are known recessive variants previously reported in the literature (13, 23, 24). The variants identified in Cases 6 and 8 are novel and suspected to be recessive. The carrier parents in both cases are asymptomatic. The paternally inherited variant in Case 8 is predicted to be damaging and is present in control populations at a low allele frequency (0.0000019) (25-27). The maternally inherited variant in Case 6 is present in control populations at a low allele frequency (0.0000012) (27) and functional studies reveal a trafficking defect typical of recessive K_ATP_ channel variants (unpublished data). Because the maternal allele is lost in the pUPD11 affected cells, the *ABCC8* variant identified in Case 6 is not expected to contribute to the HI phenotype. For these BWS–HI cases, the percentage of UPD ranged from 0% to 30% in blood, 0% to 10% in skin, and 45% to 90% in pancreas (Table 1). In addition to carrying an *ABCC8* variant, Case 7 was also heterozygous for a variant in *UCP2* that has been previously reported (28) and Case 9 was heterozygous for a previously reported MODY variant in *HNF1A* (29).

**Table 1:** Genetics table of BWS cases

Case ID	Gestational age	Birth weight (kg)		Type of BWS (chromosome 11 region of UPD, hg19)	SNP array Percentage UPD			Methylation analysis		
			HI genetics results		Blood	Skin	Pancreas	Blood	Skin	Pancreas
1	30w 6d	1.87	Neg	pUPD11 (GWpUPD)	85-95%	<5%	85%	pUPD11	n.d.	n.d.
2	38w 0d	3.75	Neg	pUPD11 (198-510-47,976,882)	0%	0%	∼85%	Neg	Neg	n.d.
3	41w 3d	4.51	Neg	pUPD11 (198,510-36,203,519)	20-25%	20-25%	80-85%	pUPD11	n.d.	n.d.
4	41w 2d	4.21	Neg	pUPD11 (198,510-14,297,872)	65%	30%	n.d.	pUPD11	n.d.	n.d.
5	36w 5d	4.16	Neg	pUPD11 (198,509-36,868,227)	80%	n.d.	n.d.	n.d.	n.d.	pUPD11
6	37w 0d	4.04	ABCC8: c.371a > g/p.Tyr124Phe (maternal)	pUPD11 (198,510-43,698,956)	25-30%	5-10%	80-90% (affected)	pUPD11	n.d.	n.d.
7	38w 1d	4.56	ABCC8: c.682g > a/p.Trp231X (paternal); UCP2: c.181g > a/p.Gly61Ser (maternal)	pUPD11 (198,510-29,902,645)	20-30%	0%	∼50%	pUPD11	n.d.	n.d.
8	38w 5d	5.95	KCNJ11: c.413t > g/p.Val138Gly (paternal)	pUPD11 (198,510-36,041,753)	0%	0%	50-55% (affected) neg (unaffected)	Neg	Neg	pUPD11 (affected); neg (normal)
9	37w 0d	5.60	ABCC8: c.3991 + 2t > c (paternal); HNF1A: c.1573a > t/p.Thr525Ser (paternal)—MODY Mutation	pUPD11 (198,510-31,027,219)	n.d.	0%	15% (unaffected) 45% (affected)	n.d.	Neg	pUPD11 (affected and unaffected)
10	37w 1d	3.91	ABCC8: c.2509c > t/p.Arg837X (paternal)	pUPD11 (198,510-47,663,049)	n.d.	0%	∼30% (unaffected) 60% (affected)	n.d.	Neg	pUPD11 (affected)
11	30w 1d	1.89	Neg	IC2 hypomethylation (none)	n.d.	0%	0%	n.d.	IC2	IC2 (affected)

Neg = negative; n.d. = not determined.

A single case of hypomethylation at IC2 without pUPD11 was identified in our cohort for this study with no other identified HI-associated mutations (Case 11; Table 1).

### Functional evaluation of BWS–HI islets

**Figure 1:**
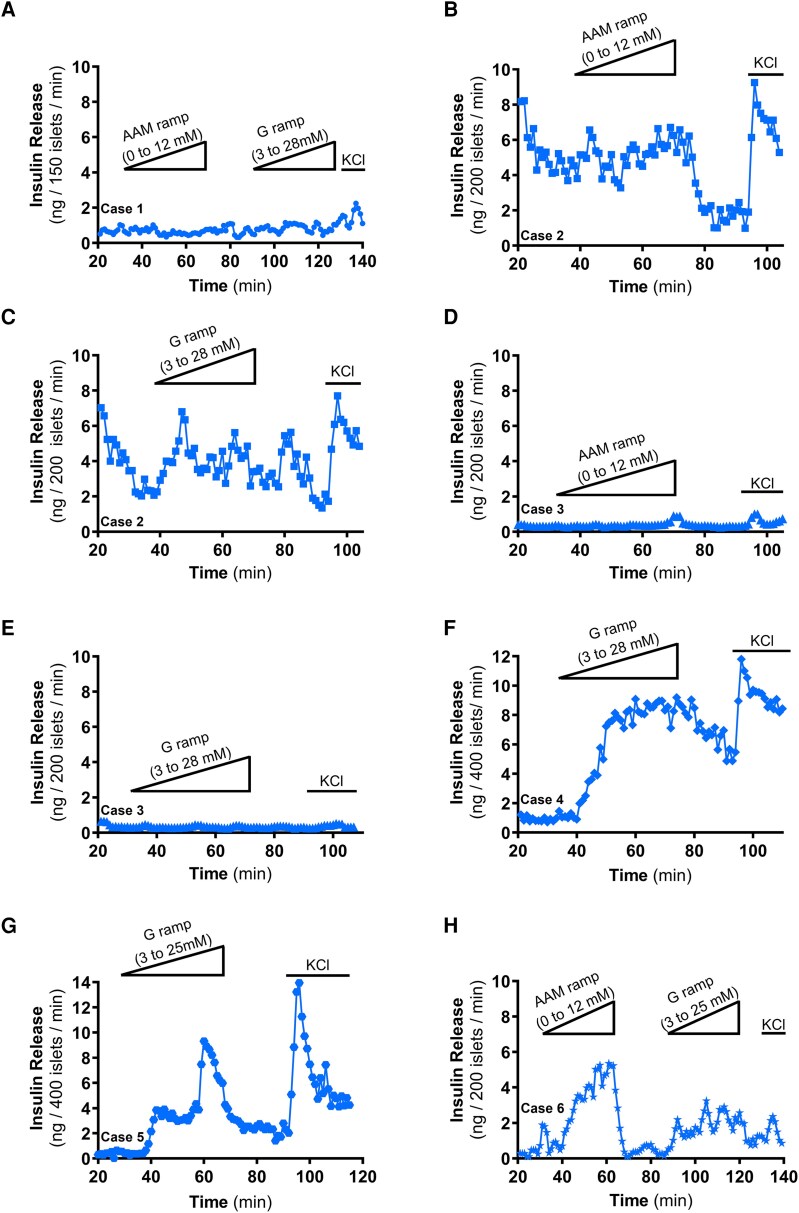
pUPD11 pancreatic islets secrete insulin in response to glucose and amino acid mixture (AAM). (A) Perifusion of pancreatic islets from Case 1 with AAM ramp (0 to 12 mM), glucose ramp (3 to 25 mM) and KCl. Perifusion of pancreatic islets from Case 2 with (B) AAM ramp, (C) glucose ramp, and KCl. Perifusion of pancreatic islets from Case 3 with (D) AAM ramp, (E) glucose ramp, and KCl. Perifusion of pancreatic islets from (F) Case 4 and (G) Case 5 with glucose ramp and KCl. (H) Perifusion of pancreatic islets from Case 6 with AAM ramp (0-12 mM), glucose ramp (3-25 mM), and KCl.

**Figure 2:**
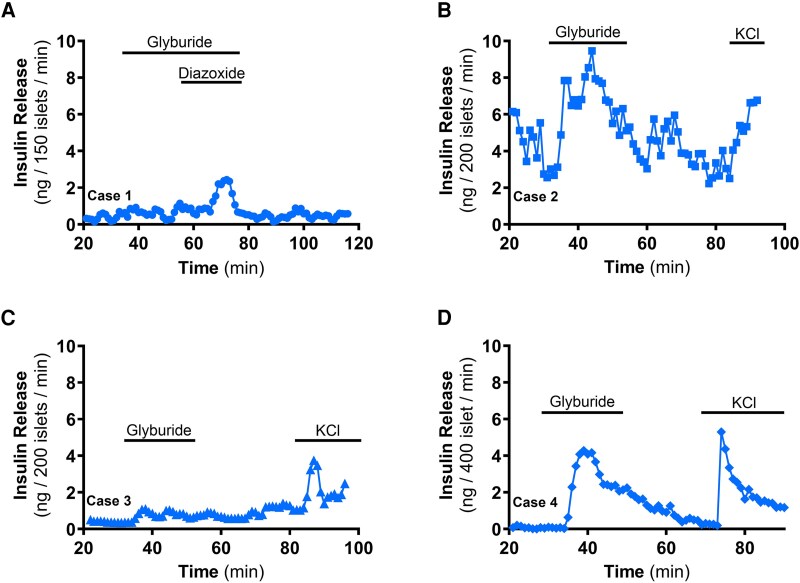
pUPD11 pancreatic islets have functional K_ATP_ channels. (A) Perifusion of pancreatic islets from Case 1 with glyburide and diazoxide. Perifusion with glyburide and KCl in pancreatic islets from (B) Case 2, (C) Case 3, and (D) Case 4.

**Figure 3:**
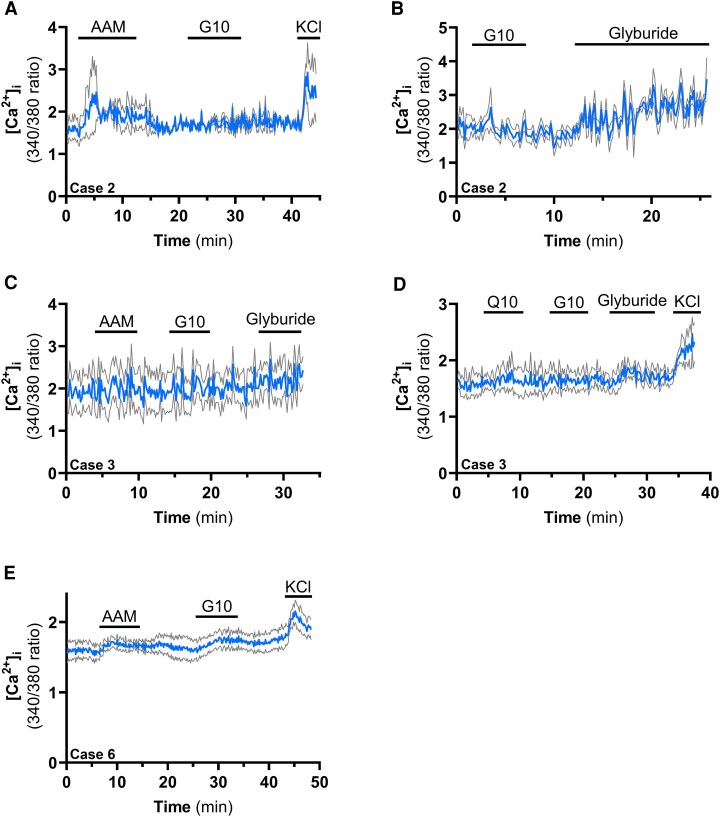
Cytosolic Ca^2+^ measurements using Fura-2AM in UPD11 pancreatic islets without a K_ATP_ mutation. For Case 2 stimulated with (A) a physiological amino acid mixture (AAM) (8 mM), glucose (10 mM), and KCl (30 mM) or (B) glucose (10 mM) and glyburide (0.6 mM). For Case 3 stimulated with (C) a physiological amino acid mixture (AAM) (8 mM), glucose (10 mM), and glyburide (0.6 mM) or (D) glutamine (10 mM), glucose (10 mM), glyburide (0.6 mM), and KCl (30 mM). (E) Case 6 islets stimulated with a physiological amino acid mixture (AAM) (8 mM), glucose (10 mM), and KCl (30 mM).

**Figure 4:**
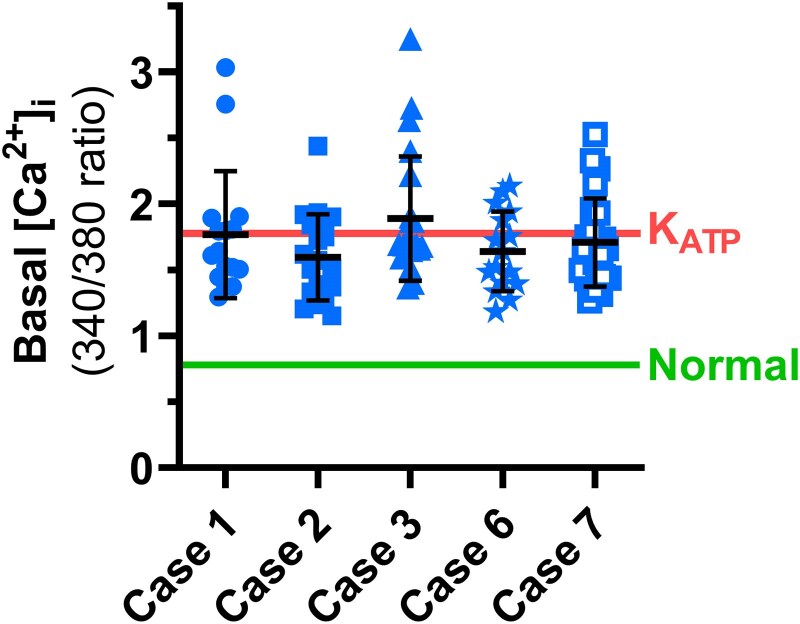
BWS–HI islets have elevated basal intracellular calcium levels [Ca^2+^]*_i_*. Basal intracellular calcium levels were measured in pancreatic islets from Cases 1, 2, 3, 6, and 7. Comparison to measurements from human normal control and K_ATP_HI pancreatic islets.

Islets from cases with pUPD11 without a predicted pathogenic K_ATP_ channel mutation (Cases 1-6), showed insulin secretion responsiveness to glucose in some but not all cases (4 of 6; Cases 2, 4, 5, and 6) and, similarly responses to stimulation with AAM were mixed with a response noted in 3 out of 4 (Cases 2, 3, and 6) cases that were tested (Fig. 1, Table 2). Of note, insulin secretion was remarkably low in response to KCl in 2 islet preparations (Cases 1 and 3), indicating that the islets were not of optimal condition (Fig. 1A, 1D, and 1E). Four of the pUPD11 islet preparations were tested for response to glyburide, with 3 (Cases 2, 3, and 4) showing a positive response (Fig. 2, Table 2). The response to AAM by islets from Case 2 and the response to glyburide by islets from Cases 2 and 3 were also appreciated by measurement of intracellular calcium [Ca^2+^]*_i_* (Fig. 3A-3D, Table 2). [Ca^2+^]*_i_* measurements in Case 6 confirm a response to both AAM and glucose (Fig. 3E, Table 2). Basal calcium measurements from the 4 pUPD11 cases evaluated (Cases 1-3, and 6) were elevated compared to controls and were equivalent to those seen in K_ATP_HI islets (18) (Fig. 4, Table 2).

**Table 2:** Functional evaluation of BWS–HI islets

		Perifusion					Cytosolic Ca^2+^ measurements				
Case ID	K_ATP_ channel mutation	Basal insulin	Glucose	AAM	Glyburide	Diazoxide	Basal	Glucose	AAM	Glyburide	Diazoxide
1*	Negative	Not elevated	−	−	−	+	Elevated	n.d.	n.d.	n.d.	n.d.
2	Negative	Elevated	+	+	++	n.d.	Elevated	−	+	+	n.d.
3*	Negative	No elevated	−	+	+	n.d.	Elevated	−	−	+	n.d.
4	Negative	Not elevated	++	n.d.	++	n.d.	n.d.	n.d.	n.d.	n.d.	n.d.
5	Negative	Not elevated	++	n.d.	n.d.	n.d.	n.d.	n.d.	n.d.	n.d.	n.d.
6	*Maternal ABCC8*	Not elevated	+	++	n.d.	n.d.	Elevated	+	+	n.d.	n.d.
7	*Paternal ABCC8*	Elevated	−	++	n.d.	n.d.	Elevated	n.d.	n.d.	n.d.	n.d.
8	*Paternal KCNJ11*	Elevated	+	−	−	−	n.d.	n.d.	n.d.	n.d.	n.d.
9	*Paternal ABCC8*	Not elevated	−	+	+	n.d.	n.d.	n.d.	n.d.	n.d.	n.d.
10	*Paternal ABCC8*	Elevated	+	++	+	n.d.	n.d.	n.d.	n.d.	n.d.	n.d.
11	Negative	Not Elevated	+	+	+	N.D.	N.D.	N.D.	N.D.	N.D.	N.D.

^*^Indicates non-optimal islet preparations as determined by weak response to KCl. n.d. indicates not determined. (−) indicates no response, (+) indicates response, (++) indicates strong response.

**Figure 5:**
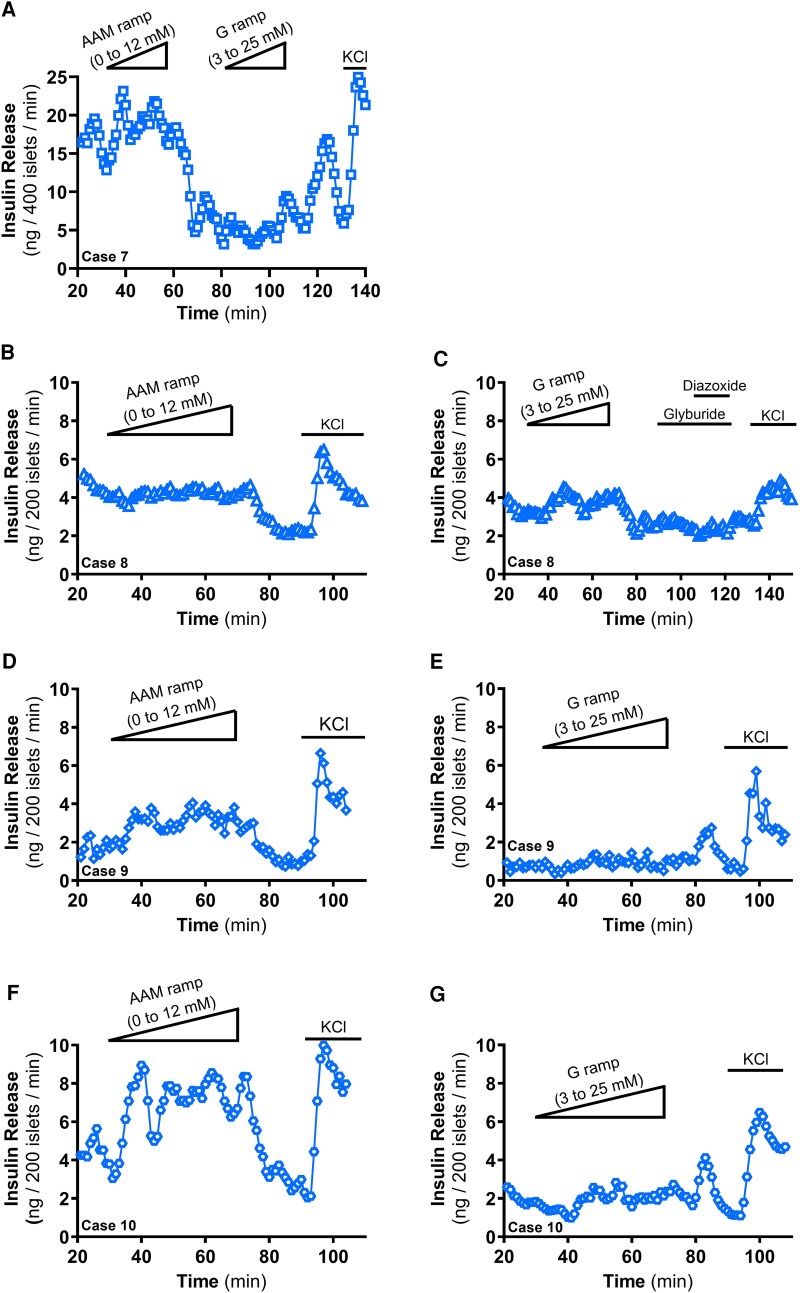
Pancreatic islets from pUPD11 with K_ATP_ channel mutations secrete insulin in response to both glucose and AAM. Perifusion of pancreatic islets from (A) Case 7 with AAM ramp (0 to 12 mM), glucose ramp (3 to 25 mM), and KCl. Perifusion of pancreatic islets from Case 8 with (B) AAM ramp with KCl and (C) glucose ramp, glyburide, diazoxide, and KCl. Perifusion of pancreatic islets from (D, E) Case 9 and (F, G) Case 10 with AAM ramp with KCl and glucose ramp with KCl.

**Figure 6:**
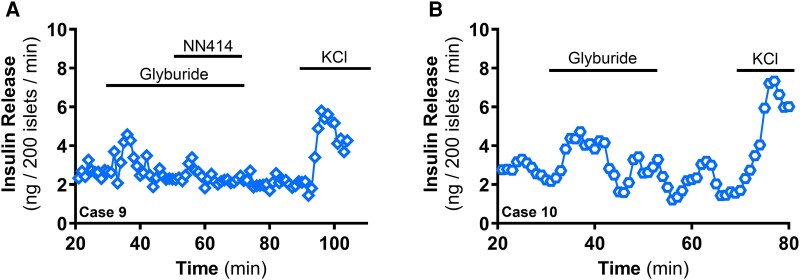
pUPD11 pancreatic islets with K_ATP_ channel mutations respond to glyburide treatment. (A) Perifusion of pancreatic islets from Case 9 with glyburide, NN414, and KCl. (B) Perifusion of pancreatic islets from Case 10 with glyburide and KCl.

Islets from the pUPD11 cases with predicted pathogenic K_ATP_ channel mutations showed heterogeneous fuel responsiveness. Three out of 4 of these cases (Cases 7, 9, and 10) secreted insulin in response to stimulation with AAM to varying degrees and 4 out of 4 cases secreted insulin in response to stimulation with glucose (Cases 7–10; Fig. 5). Though of note, Case 9 only showed a mild response to glucose that just met the minimum threshold of 50% increase from baseline. Of the 3 islet preparations tested, 2 demonstrated insulin secretion in response to glyburide (Cases 9 and 10), indicating the presence of some functional K_ATP_ channels (Figs. 5D and 6). Measurements of basal calcium of Case 7 showed significantly increased levels compared to normal controls and comparable to that seen in islets isolated from patients with K_ATP_HI (Fig. 4).

**Figure 7:**
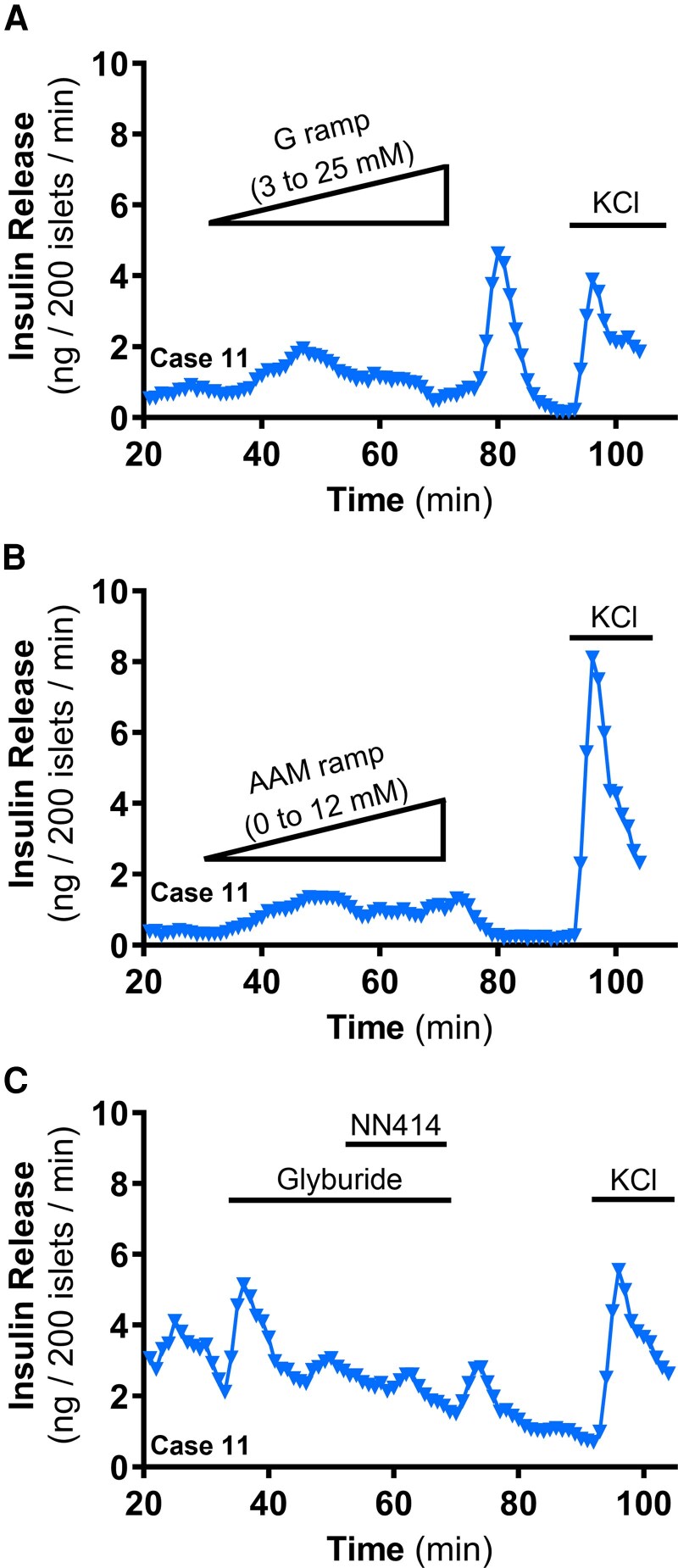
Pancreatic islets with hypomethylation at IC2 are responsive to glucose, AAM, and glyburide. Perifusion of pancreatic islets from Case 11 with hypomethylation at IC2 with (A) glucose ramp with KCl, (B) AAM ramp with KCl, and (C) glyburide, NN414, and KCl.

Functional evaluation by perifusion of islets isolated from the case with hypomethylation at IC2 revealed stimulated insulin secretion in response to both glucose and AAM (Fig. 7). The islets were responsive to glyburide indicating functional K_ATP_ channels, but, interestingly, we did not observe a response to the K_ATP_ channel opener, NN414 (aka tifenazoxide; Fig. 7).

### RNA-seq analysis of BWS pUPD11 islets

**Figure 8:**
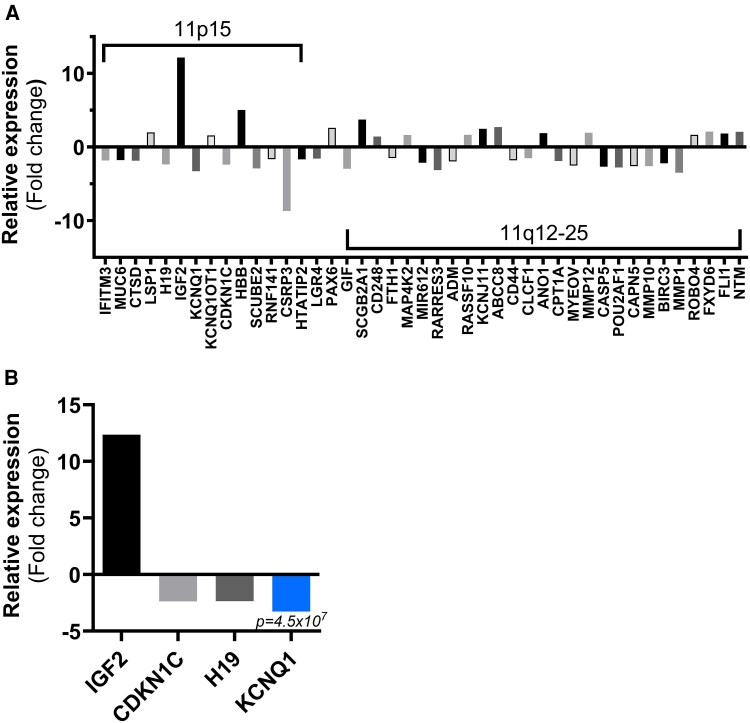
Expression of loci within the chromosome 11p15 region demonstrates the greatest change in BWS–HI islets. (A, B) Relative expression of genes on Chromosome 11 via RNAseq.

To investigate the potential causative mechanism of hyperinsulinism in BWS, we performed gene expression analyses in islets isolated from children with BWS–HI. RNA sequencing analysis was completed on pancreatic islets isolated from patients with mosaic pUPD11 of chromosome 11p15.5 and compared to that of islets isolated from unaffected pancreatic tissue from age-matched controls who underwent pancreatectomy for focal HI. Analysis of genes within chromosome 11 revealed that the most significant expression changes occurred within the BWS-associated chromosomal 11p15 region. As expected, the largest observed increase in expression was the paternally imprinted insulin-like growth factor 2 (*IGF2*) by 12-fold, while a 2-fold decrease in expression was observed in the maternally imprinted genes cyclin-dependent kinase inhibitor 1C (*CDKN1C*) and *H19* (Fig. 8A). These changes in gene expression offer insight into the mechanism underlying the increased beta cell mass observed in BWS–HI, but do not explain the functional changes in insulin secretion. An interesting candidate gene for insulin secretion in this region is *KCNQ1*, which encodes the pore-forming alpha subunit of the voltage-gated K^+^ channel, Kv7.1. Clinically, changes in KCNQ1 activity have been linked with changes in insulin levels (30, 31). Our results demonstrate a 3-fold decrease in *KCNQ1* expression in BWS–HI islets compared to controls (Fig. 8B).

## Discussion

It has previously been postulated that hyperinsulinism in BWS is the result of overgrowth in beta cell mass; however, while the increase in endocrine tissue may play a part in the overall volume of insulin secreted, our results in BWS–HI pancreatic islets indicate that there are distinct functional changes that cannot be explained by increased beta cell mass alone. Previously, it was established that beta cell mass is highly variable in the human population, with up to a 2-fold difference observed between normal individuals, suggesting that increased mass can be normal for some individuals without HI (32, 33). There are also conditions, such as pregnancy, in which beta cell mass increases by up to 2-fold with no resulting HI (34). In healthy individuals with appropriately functioning beta cells, insulin secretion is tightly regulated according to plasma glucose concentrations, but, in HI, genetic changes in genes important for the regulation of insulin secretion lead to dysregulated insulin secretion that is not dependent on plasma glucose concentration (35). We previously showed that pancreatic islets isolated from infants with inactivating mutations in the K_ATP_ channel genes exhibit higher baseline cytosolic calcium and insulin secretion, impaired glucose-stimulated insulin secretion, and increased insulin secretion in response to stimulation with a physiologic mixture of amino acids (18). These features of the K_ATP_-HI islets are consistent with the phenotypes observed in patients with K_ATP_-HI, which is characterized by fasting hypoglycemia, impaired glucose tolerance (36), and protein-induced hypoglycemia (37).

The study of islets from infants with BWS–HI demonstrates similarities of the islet phenotype with that of K_ATP_-HI islets, specifically elevated concentrations of intracellular Ca^2+^ and secretion of insulin in response to stimulation with amino acids (Figs. 1, 4, 5 and 7, Table 2). Though unlike K_ATP_HI islets, BWS–HI islets largely retain responsiveness to stimulation with glucose and the K_ATP_ channel inhibitor glyburide (Figs. 1, 2, 5, 6 and 7, Table 2). It is important to note that, even with respect to HI, there can be a spectrum of phenotypes in BWS and strides must be taken to identify the causative etiology in the context of any additive mutations to evaluate this range of manifestations better. The broad phenotypic spectrum and the large swath of loci affected by genetic and epigenetic abnormalities in BWS have made it challenging to identify the specific cause of hyperinsulinism. Yet, one commonality across all cases in this cohort is the loss of *KCNQ1* expression in pancreatic islet cells.

Insulin release from beta cells is closely regulated by the contingent of ion channels in the cell membrane. The importance of ion channels in insulin release is well evidenced by the disease states involving mutations of β-cell ion channels, such as K_ATP_. Specifically, inactivating and activating mutations in the K_ATP_ channel are associated with hyperinsulinism (K_ATP_HI) and neonatal diabetes mellitus, respectively (38-40). Our expression analysis (Fig. 8) indicates that another K^+^ channel, Kv7.1, is a viable candidate for regulating insulin secretion candidate and warrants further examination. Furthermore, inactivating mutations of *KCNQ1* result in long QT type 1 with postprandial hyperinsulinemia and reactive hypoglycemia, while activating mutations of *KCNQ1* lead to glucose-stimulated hypoinsulinemia (30, 31). Therefore, we posit that the lack of *KCNQ1* activity plays a key role in the pathophysiology of hyperinsulinism in BWS.

The current study has some limitations that should be acknowledged. First, islets for the study were obtained from surgical specimens processed in pathology prior to the isolation procedure, which may have compromised their quality. Indeed, the poor response to KCl observed with islets from cases 1 and 3, suggests suboptimal islet quality. Second, because of the mosaic nature of genetic and epigenetic changes in BWS, it is likely that some of the islets included in the perifusions may be normal, explaining the heterogeneity of the response. Indeed, evidence of K_ATP_ channel function in cases 9 and 10, which would be predicted to lack K_ATP_ channels expressed because of pUPD11 and paternal recessive inactivating *ABCC8* variant, suggests the presence of some normal ß-cells. Lastly, our gene expression studies used whole islets rather than ß-cells, which may have reduced the magnitude of the observed changes.

Despite these limitations, the insights derived from the study of islets isolated from infants with BWS–HI are extremely valuable and demonstrate the complex pathophysiology responsible for the hyperinsulinism in this condition.

## Data Availability

Some or all datasets generated during and/or analyzed during the current study are not publicly available but are available from the corresponding author on reasonable request.
